# Origin and Diversification of TRIM Ubiquitin Ligases

**DOI:** 10.1371/journal.pone.0050030

**Published:** 2012-11-21

**Authors:** Ignacio Marín

**Affiliations:** Instituto de Biomedicina de Valencia (IBV-CSIC), Consejo Superior de Investigaciones Científicas, Valencia, Spain; Ecole Normale Supérieure de Lyon, France

## Abstract

Most proteins of the TRIM family (also known as RBCC family) are ubiquitin ligases that share a peculiar protein structure, characterized by including an N-terminal RING finger domain closely followed by one or two B-boxes. Additional protein domains found at their C termini have been used to classify TRIM proteins into classes. TRIMs are involved in multiple cellular processes and many of them are essential components of the innate immunity system of animal species. In humans, it has been shown that mutations in several TRIM-encoding genes lead to diverse genetic diseases and contribute to several types of cancer. They had been hitherto detected only in animals. In this work, by comprehensively analyzing the available diversity of TRIM and TRIM-like protein sequences and evaluating their evolutionary patterns, an improved classification of the TRIM family is obtained. Members of one of the TRIM subfamilies defined, called Subfamily A, turn to be present not only in animals, but also in many other eukaryotes, such as fungi, apusozoans, alveolates, excavates and plants. The rest of subfamilies are animal-specific and several of them originated only recently. Subfamily A proteins are characterized by containing a MATH domain, suggesting a potential evolutionary connection between TRIM proteins and a different type of ubiquitin ligases, known as TRAFs, which contain quite similar MATH domains. These results indicate that the TRIM family emerged much earlier than so far thought and contribute to our understanding of its origin and diversification. The structural and evolutionary links with the TRAF family of ubiquitin ligases can be experimentally explored to determine whether functional connections also exist.

## Introduction

Present in all eukaryotic organisms, ubiquitination is involved in multiple essential functions. It has a critical role regulating protein levels: the addition of a polyubiquitin chain often targets a protein for degradation by the proteasome. However, ubiquitination has many other important tasks which often do not involve the degradation of the tagged proteins. This versatility allows for many facets of cell signaling, endocytosis, DNA repair or gene expression, among other cellular processes, to be controlled by ubiquitination [1]–[6]. The most diverse components of the ubiquitination system are ubiquitin ligases (E3s), the group of enzymes able to transfer ubiquitin to target proteins. E3s, which provide specificity to the machinery, are often very numerous, with many species (e.g. humans) having hundreds of genes encoding them. E3s are classified into a few classes, depending first on whether they are single proteins or form multiprotein complexes and second, on structural and functional features of the proteins themselves [1]. We have recently studied the evolution of several of the most important classes of ubiquitin ligases, including cullin-containing E3 complexes [7], HECT domain-containing ubiquitin ligases [8] and U-box E3s [9]. However, the most diverse E3s are those that contain RING fingers, either alone or in combination with other protein domains [10]. The analysis of that class of proteins as a whole is difficult, given that their only common feature is the RING finger itself. This is a relatively small and rapidly evolving domain that does not provide enough information as to allow for the evolutionary characterization of the relationships among the different RING-containing ubiquitin ligase types. In the last years, we have extensively studied the diversification of a particular group of RING finger E3s, called RBR (Ring – Between Rings – Ring) family (reviewed in [11]). We focused on RBR proteins because they contain a unique RING – IBR – RING supradomain that makes feasible precise evolutionary analyses [11]–[15]. Significantly, this RBR-specific structural feature correlates with the fact that they perform ubiquitination differently from typical RING-only E3s, in a way that partially resembles HECT E3 function [16].

Ubiquitin ligases of the TRIM family (a. k. a. RBCC family; see [17]–[21] for reviews) are another type of RING-containing E3s that share complex structural features, allowing detailed evolutionary studies. In addition of an N-terminal RING finger domain, TRIM E3s typically contain one or two B-boxes, short domains probably derived from the RING finger [22], which are only found in this family. The B boxes are located C-terminally with respect to the RING finger. After the B box(es), TRIMs often also have a coiled coil (CC) domain. This RBCC (RING – B box – [B box] – CC) supradomain is sufficiently long and conserved as to provide useful phylogenetic information, as shown in several significant previous works [23]–[25]. Sardiello *et al.* [23] focused their analyses on characterizing the orthologs of human TRIM genes in some vertebrate and invertebrate model species, concluding that the TRIM family can be divided into two main groups, one of them (“Group 1”) present in both vertebrates and invertebrates and structurally very diverse and the other (“Group 2”) restricted to vertebrates and characterized by all proteins in that group containing a SPRY domain, which is thought to be involved in facilitating protein-protein interactions [26]. However, some of the proteins that belong to the Group 1 defined by [23] also have SPRY domains [23], [27]. The study by Van der Aa *et al.* [24] was devoted to analyze the rapid amplification, linked to changes in their selective regimes, of particular SPRY-containing TRIMs in fishes. Additional evidence for rapid evolution and increase of complexity of SPRY-containing TRIM E3s in fishes has been recently obtained [25].

From a functional point of view, TRIM E3s have been extensively studied. It is known that mutations in several human TRIM genes cause genetic diseases such as mulibrey nanism (*TRIM37* mutations; [28]), Opitz syndrome (mutations in *TRIM18/MID1*, where the slash separates two alternative names used for a particular gene or protein; [29]), muscular dystrophy limb-girdle Type 2H (*TRIM32*; [30]), Bardet-Biedl syndrome 11 (also *TRIM32*; [31]–[32]) and familial mediterranean fever (*TRIM20/MEFV*; [33]–[35]) and may contribute to many other, including autoimmune and inflammatory diseases [36]–[38], muscular dystrophies (e.g. [39]), neurodegenerative diseases [40]–[41] or multiple types of cancer [42]–[50]. In addition, several TRIM proteins are key actors in innate immunity responses, especially down-regulating the replication of many different types of viruses [51]–[73]. It is also significant that, although it has been shown already that about 20 different TRIM proteins act as ubiquitin ligases, at least one of them (TRIM25/EFP) functions also as a ISG15 ligase [74] and several may act as SUMO ligases [75]–[76].

Despite the great interest aroused by TRIM proteins, leading to hundreds of papers published on members of this family, no attempt for a systematic evolutionary analysis of all TRIMs present in eukaryotes has been ever attempted. This may lead to significant shortcomings in our understanding of this family. For example, the generally accepted classification of TRIM proteins into classes [20], [77], widely used as reference in functional studies, is exclusively based on structural features, i.e. the presence or absence of some protein domains. This type of classification rests on the idea that the acquisition of a protein domain is a sufficiently infrequent event as to be considered a unique phylogenetic marker. If this is strictly true, the defined classes would be monophyletic, with all the genes encoding proteins of a particular class deriving from a common ancestor. Following this strategy, the existence of nine distinct TRIM classes were suggested by Short and Cox [77]. Later, this classification was slightly modified by Ozato *et al.* [20] using additional data, leading to the definition of eleven classes (C-I to C-XI). However, without extensive phylogenetic analyses to support the monophyly of these classes, it is impossible to ascertain whether they are indeed natural groups. For example, convergence, in which a protein domain is acquired two or more times independently by unrelated members of a protein family, is a common occurrence, and has been found in other ubiquitin ligase families (e.g. [8], [12]). It is also significant that the classifications provided by Short and Cox [77] and Ozato *et al.* [20] fit poorly with the suggestions of Sardiello *et al.* [23], which are based on phylogenetic analyses of a small but still significant sample of TRIM sequences. Particularly, the suggestion of dividing TRIMs into just two groups and putting together in a single group (“Group 1”) a large number of structurally very diverse TRIM proteins [23] was in radical contradiction with the domain-based classifications. Choosing one or the other view may significantly influence how the functional analyses of TRIM genes and proteins are tackled and interpreted.

In this work, a complete phylogenetic analysis of the TRIM family is described. The focus of this study is twofold. First, to establish the origin of the TRIM family, confirming whether or not it is restricted to animals. Second, to provide an account of the early patterns of diversification of the family in order to refine, if necessary, its current classification. As it will be shown in the next sections, several unexpected results were found, the main ones being that TRIM proteins most likely emerged very early in eukaryotic evolution and that they are potentially related to a different type of RING finger-containing ubiquitin ligases known as Tumor necrosis factor-Receptor Associated Factors (TRAFs). Phylogenetic data generally agree with the domain-based classification [20], [77], although some discrepancies were detected. Also, the origin and evolution of several TRIM-like proteins, so far never systematically studied, is analyzed.

## Materials and Methods

To generate a comprehensive database of TRIM proteins, Tblastn searches were performed, using multiple representative TRIM family sequences as queries and with default parameters, against the non-redundant, htgs, gss, est and wgs databases of the National Center for Biotechnology Information (http://www.ncbi.nlm.nih.gov/). The TRIM query sequences were selected from all the classes defined by Short and Cox [77] and Ozato *et al.* [20], in order to detect the whole range of TRIM sequence variation. From these Tblastn searches, all the proteins with high similarity to the queries were selected. Both a very low Expect (E) value (typically, E<10^−10^) and a minimal size (S) of the region showing similarity to the query sequences were demanded to classify a protein as a positive hit. In general, S was required to be larger than 300 amino acids. This cutoff was lowered to S = 180–200 amino acids when the query protein sequence was very short. The sets of sequences obtained in each search were aligned using Clustal X 2.0.12 [78] amd MAFFT v6.864.b [79] with default parameters. These results were compared to establish a consensus alignment and manually corrected using GeneDoc 2.7 [80]. From the final protein alignments, preliminary phylogenetic trees were obtained using the neighbor-joining (NJ) method implemented in the MEGA 5.0.5 program [81]. These trees were used to evaluate the congruence between the original structural classes defined by Short and Cox [77] and Ozato *et al.* [20] and the groups obtained by sequence similarity. Three types of discrepancies were observed: 1) In a few cases, sequences of proteins of a given structural class were found in two different trees, in one of them together with very similar proteins of the same structural class and in a second one, with proteins of a different structural class, appearing then as highly divergent sequences, with long branches. This was simply due to some similarities among proteins of different classes being above the conventional E and S values used as thresholds. These duplications were solved considering that the structural and sequence-based analyses were congruent and eliminating the sequences from the tree in which they were lumped together with divergent sequences of a different structural class; 2) In some other cases, proteins classified as belonging to two different structural classes indeed had very similar sequences – as similar as proteins within the same class – appearing together in a given tree. These cases were interpreted as showing that the structural data were incongruent with the sequence data, suggesting that the classification of those proteins had to be modified. All these cases will be detailed below, in the Results section; 3) A final type of discrepancy was the finding of proteins with very high similarity to TRIMs but that could not be classified as bona fide TRIM proteins, given that they did not have complete RBCC supradomains. These TRIM-like proteins are also discussed in detail in the next section.

**Table 1:** Classification of TRIM and TRIM-like proteins (these last ones, between parenthesis).

SUBFAMILY	Structural class (Ozato et al. 2008. Ref. [20])	Phylogeneticrange	TRIM (TRIM-like) human, mouse genes	TRIM (TRIM-like)*Drosophila*genes	TRIM (TRIM-like)*Caenorhabditis*genes
A	VIII	Eukaryotes	*TRIM37*	–	–
B	I, II	Animals	*TRIM1/MID2, TRIM9, TRIM18/MID1,* *TRIM36, TRIM46, TRIM54/MURF3,* *TRIM55/MURF2, TRIM63/MURF1, TRIM67,* *TRIM76/CMYA5, (FSD1), (FSD2), (FSD1L)*	*Trim9/CG31721*	*Madd-2*
C	VII, X	Animals	*TRIM2/NARF, TRIM3/BERP,* *TRIM32, TRIM45, TRIM56, TRIM71,* *(NHLRC1/MALIN)*	*Mei-P26, Abba* *(Wech/CG1624), (Brat)*	*Nhl-2, Ncl-1, Lin-41, Nhl-3,* *(Nhl-1)*
D	IX	Cnidarians,bilaterians	*TRIM23/ARD1*	*–*	*Arc-1*
E	VI	Bilaterians	*TRIM24/TIF1-ALPHA, TRIM28/TIF1-BETA,* *TRIM33/TIF1-GAMMA,* *TRIM66/TIF1-DELTA*	*Bonus*	*–*
F	–	Bilaterians	*(RNF207)*	*–*	*(F4769.4)*
G	IV	Vertebrates	*TRIM4, TRIM5, TRIM6, TRIM7/GNIP,* *TRIM10/HERF1, TRIM11, TRIM12, TRIM15,* *TRIM16/EBBP, TRIM17/TERF, TRIM21/RO52,* *TRIM22/STAF50, TRIM25/EFP, TRIM26, TRIM26-LIKE, TRIM27/RFP, TRIM30, TRIM30A,* *TRIM31, TRIM34, TRIM35/HLS5, TRIM38/RORET,* *TRIM39, TRIM41/RINCK, TRIM43-LIKE, TRIM47/GOA, TRIM47-LIKE,* *TRIM48, TRIM49/RNF18, TRIM49B, TRIM49L2, TRIM50,* *TRIM51/SPRYD5, TRIM52, TRIM53, TRIM58, TRIM60/RNF33,* *TRIM61/RNF35, TRIM62/DEAR1, TRIM65, TRIM68/SS56, TRIM69/RNF36,* *TRIM72/MG53, TRIM73, TRIM74, TRIM75, TRIM77, TRIML1, TRIML2, LOC283116,* *LOC440011, LOC120824, LOC283116, LOC100134006*	*–*	*–*
H	XI	Vertebrates	*TRIM13/RFP2, TRIM59, MRF1*	*–*	*–*
I	III	Birds, Mammals	*TRIM42*	*–*	*–*
Additional TRIM genes, vertebrates(Subfamilies J–Q)	V/−	Restricted to some/all vertebrates	*TRIM8/GERP, TRIM14/PUB, TRIM19/PML,* *TRIM20/MEFV, TRIM29/ATDC,* *TRIM40, TRIM44, (BSPRY)*	*–*	*–*
Additional TRIM genes, *Drosophila*	–	Drosophilids	*–*	*CG8419, CG14306*	
Additional TRIM genes, *Caenorhabditis*	–	*Caenorhabditis* genus	*–*	*–*	*ZK945.4, (C28G1.6), (B0281.3), (B0281.8), (ZK1240.5), (F43C11.8), (ZK1240.1), (ZK1240.2), (C28G1.5), (ZK1240.9), (F43C11.7), (ZK1240.8), (ZK1240.3), (ZK1240.6)*

Genes in selected model species are indicated.

At this point, the diversity of TRIM sequences in human, mouse, *Drosophila melanogaster* and *Caenorhabditis elegans* was explored in full, to determine whether all proteins in those species were present in the alignments and trees obtained. Given that the TRIM family members of these model species have been studied in great detail before [23], these specific analyses served to determine whether the simple strategy outlined above was indeed sufficient to detect all the TRIM sequences present in the databases, even the most divergent ones. The conclusion was that more than 95% of the known sequences were unearthed by the Tblastn-based analyses. The rest, which were all small, highly divergent TRIMs lacking any protein domain other than the RING and B-box(es), were separately analyzed, one by one, to establish the presence of orthologs in other species. Most of these outlayer TRIMs were restricted to just a few closely-related species, what contributes to explain why they were not detected in the standard Tblastn searches. After these additional searches were finished, the database generated contained 1952 TRIM protein sequences, divided into the groups found, which will be called subfamilies from now on. These subfamilies included from 12 sequences (e.g. divergent proteins found just in a few species) to 736 sequences (a large subfamily with many genes present in a large number of organisms). A final check showed that proteins of a given subfamily were always much more similar, as shown by very low Tblastn Expect values (10^−22^≥E≥0, with most proteins having E≤10^–29^) than proteins of different subfamilies (E≥10^−16^, but often E>>10^−10^).

**Figure 1:**
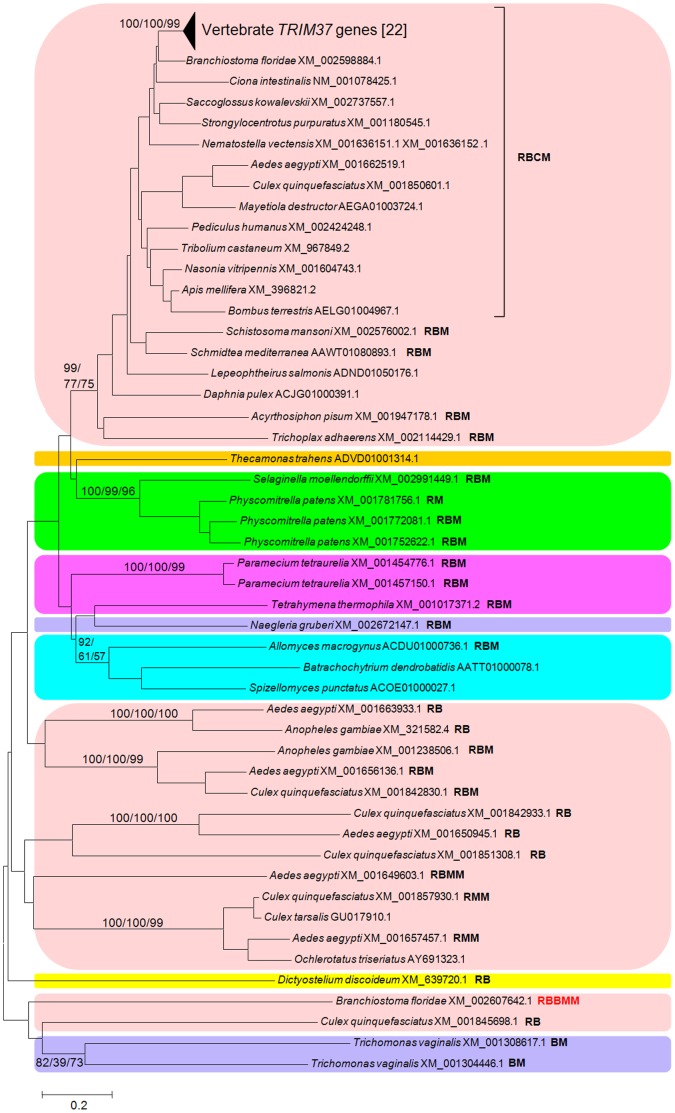
Dendrogram including all Subfamily A protein sequences. Species and accession numbers are indicated. Numbers above the branches indicate percentage of support, according to bootstrap analysis, ordered as NJ/MP/ML (see Methods). For simplicity, only external branches with significant boostrap values are detailed. Colors indicate the phylogenetic range, in order from top to bottom: animals (pink), apusozoans (orange), plants (green), alveolates (magenta), excavates (violet), fungi (blue) and amoebozoa (yellow). In capital, bold letters the protein structures are summarized, according to InterProScan searches (R: ring finger; B: B box; C: B-box C terminal domain, M: MATH domain). It was not possible to characterize the structures of several of these proteins, for which only partial sequences were available. It can be deduced that the ancestral structure was RBM, with several derivative structures (e.g. RBCM, RBMM, RMM) or losses of domains (RB, BM, RM) occurring in the proteins of particular groups or species. A single *Branchiostoma floridae* sequence, discussed in the text, had a RBBMM structure (red).

From this final database, the species range of all the TRIM-encoding genes was determined. Only one group of TRIM proteins (that will be called Subfamily A throughout this text) was found to be present not only in metazoans but in other eukaryotes (see Results). Precise Subfamily A-specific phylogenetic trees were obtained following methods already described in previous works of our group. In brief, dendrograms were obtained using three different methods of tree reconstruction, neighbor-joining [NJ], maximum parsimony [MP] and maximum likelihood [ML]. The NJ and ML trees were obtained using the routines in MEGA 5.0.5 [80] while MP analyses used PAUP* beta 10 version [82]. For NJ, sites with gaps were treated with the pairwise deletion option, as recommended in [83], and Kimurás correction was used. Parameters for MP were as follows: 1) all sites included, gaps treated as unknown characters; 2) randomly generated trees used as seeds; 3) maximum number of trees saved equal to 100; and, 4) heuristic search using the subtree pruning-regrafting algorithm. Finally, for ML analyses, the BioNJ tree was used to start the iterative searches and it was determined, with MEGA 5.0.5, that using the JTT matrix with variation in rates among sites according to a gamma distribution with four discrete categories and a fraction of invariable sites was the best way to model the amino acidic substitutions. Gaps were also treated as unknown characters. The close-neighbor interchange routine was used to explore the landscape of ML trees. Reliability of the topologies was tested in all cases by bootstrap analyses. For Subfamily A analyses, which included a relatively small number of sequences, 1000 bootstrap replicates were performed for the NJ and MP analyses and 200 for the ML analyses, which are much more computer intensive. Given the relationship found between the most ancient TRIM sequences and TRAF sequences (see below), phylogenetic analyses of MATH domain-containing proteins were made using the same methods described above. However, given the size of this dataset (517 sequences), the number of bootstrap replicates was reduced in both the MP analyses (200 replicates) and in the ML analyses (100 replicates). The domains present in TRIM and MATH-containing proteins were characterized using InterProScan [84]. Dendrograms were drawn using the tree editor of MEGA 5.0.5.

**Figure 2:**
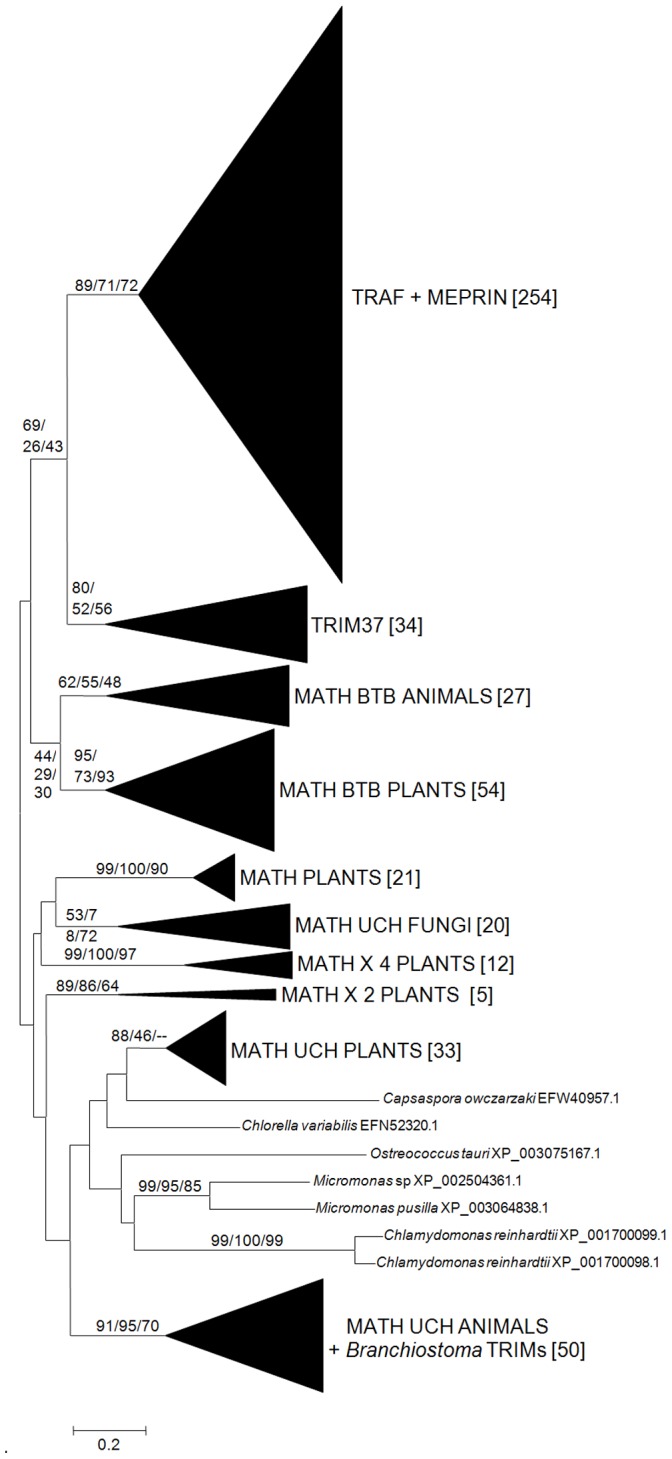
Dendrogram obtained for MATH-containing proteins. Numbers along the branches again refer to NJ/MP/ML bootstrap support. In brackets, the number of proteins within each class. TRAFs and Meprin proteins appeared together in the trees, as did MATH UCH animal proteins (a type of ubiquitin proteases) with a few *Branchiostoma* Subfamily C TRIMs (discussed in the text). Plant MATH, MATH×2 and MATH×4 groups correspond to sequences that only have one, two or four MATH domains, respectively. Seven sequences of uncertain classification are indicated in full. See [85] for additional details of all these groups.

## Results

### Comprehensive Sequence Analyses Improve the Classification of the TRIM Family

As indicated in the Introduction section, whether the domain-based classification [20], [77] is confirmed using evolutionary analyses had not hitherto been explored in detail. By considering the results of the precise searches and phylogenetic analyses described in the Material and Methods section, it is relatively easy to tackle this question. Once all TRIM proteins were classified into subfamilies according to sequence analyses, it could be established when those groups did not agree with the structure-based classes. This leads to a refined classification of TRIM proteins, detailed in Table 1. The comparative sequence analyses indicated that most TRIM proteins can be naturally classified into nine main subfamilies, which are named in Table 1 with a letter (A–I). That letter was assigned according to how wide is the phylogenetic range of species in which these subfamilies are detectable. Therefore, given that phylogenetic range and age are generally correlated, Subfamily A is expected to be the oldest and Subfamily I is the most recently emerged. Actually, it was found that Subfamily A is so old that predates the origin of the metazoans, an important result that is described in detail in the next section.

In Table 1, the parallelism between this new classification and the domain-based ones is shown and the genes in mammals, flies and nematodes assigned to the different subfamilies are detailed. A first conclusion drawn from the comparison of both classifications is that they generally agree. As expected, proteins that are similar enough as to be grouped in the sequence-based searches generally share also common domains. However, several differences became also apparent (see Table 1). The first one is that the sequences of proteins of two of the classes defined by Short and Cox [77] and Ozato *et al.* [20], namely Class I and Class II, are extremely similar and thus can be naturally grouped into a single subfamily (Subfamily B; Table 1). This result was also found by Sardiello *et al.* [23]. The most parsimonious hypothesis compatible with the available data is that Class II genes are truncated duplicates of typical Class I genes. Although Class II proteins lack the N-terminal domains characteristic of Class I proteins, such as Fibronectin type 3 or SPRY domains [77], these differences can be simply explained by recent losses of these domains, in genes that are restricted to vertebrate species. A second difference is that Class V, defined by those authors as containing all TRIM proteins that lack any obvious domain besides the RBCC supradomain, is clearly not monophyletic. On the contrary, six of the proteins included in that class (TRIM31, TRIM52, TRIM56, TRIM61/RNF35, TRIM73 and TRIM74) are very similar in sequence to proteins in other classes, and therefore it may be assumed once again that they derived from duplications followed by domain losses. The genes encoding these six proteins can be assigned to Subfamilies C (*TRIM56*) and G (the other five). Respect to the rest of proteins that were included in Class V, no clear sequence similarities with proteins in subfamilies A–I or among them were detected and therefore it is unclear how to classify them. In Table 1, the genes encoding these proteins, plus some that also lack any additional domain besides those found in the RBCC signature but were not included in any class by Short and Cox [77] or Ozato *et al.* [20], are put together as “additional TRIM genes”. Alternatively, it is possible to assign each of these genes to a different subfamily, which may be named as follows: J (for *TRIM8/GERP* genes), K (*TRIM14/PUB*), L (*TRIM19/PML*), M (*TRIM20/MEFV*), N (*TRIM29/ATDC*), O (*TRIM40*), P (*TRIM44*) and Q (*BSPRY*). Additional, divergent genes that cannot be classified in subfamilies A–Q were found in the invertebrate model species analyzed (see details in Table 1).

A third difference of the sequence- and structure-based classifications is that the former allows to detect several genes that are clearly related in sequence to canonical TRIM proteins, but lack part of the basic RBCC signature, normally either the RING finger or both B boxes. The genes encoding these “TRIM-like” proteins (between parentheses in Table 1) can be hypothesized to derive from typical TRIM genes, again by duplications followed by protein domain losses. Many of these genes are relatively recent in evolutionary terms, although a few (e.g. *RNF207*, which defines Subfamily F) are quite ancient. A fourth, final discrepancy is that class X, defined by Ozato *et al.* [20] by separating TRIM45 from the rest of Class VII proteins based on the presence of a filamin domain in TRIM45, does not seem warranted. As first suggested by Short and Cox [77], it is more natural to put them all together in a single group (Subfamily C), given their high similarity.

### Subfamily A Genes are Present in Many different Eukaryotic Groups

Subfamily A proteins, which correspond to Class VIII in the domain-based classification [20], [77], contain an additional C-terminal domain called MATH [85] (in some databases, it receives the alternative name of TRAF domain). The MATH domain is involved in facilitating protein-protein interactions [86] and appears in just a few protein families, as will be detailed in the next section. In humans, there is a single Subfamily A gene, *TRIM37*. Surprisingly, genes very similar both structurally and in sequence to human *TRIM37* were found not only in animals but also in many other types of organisms, including a few fungi and plants and several, diverse groups of protozoans (Figure 1; see details of the taxonomy and protein structures in that figure). This was quite surprising, given that TRIM genes were hitherto assumed to exist only in animals. These new results show that this is not the case, indicating that the TRIM family is much older than previously thought. Figure 1 also shows that these searches detected a lineage-specific amplification of *TRIM37*-like genes in mosquito species. These genes encode proteins with 0 to 2 MATH domains. Also, a single *TRIM37*-like gene in *Dictyostelium discoideum* (which encodes a protein lacking the MATH domain) and two in *Trichomonas vaginalis* (encoding proteins lacking RING finger) were discovered. Finally, a single exceptional, very divergent gene was found in the *Branchiostoma floridae* genome that apparently not only encodes for a protein with two MATH domains, but also with two B boxes. This was unexpected, given that all the rest of Subfamily A proteins only have one B box. It is unlikely this putative gene is a genomic assembly artifact, because it is found in the *Branchiostoma* genome as a short, intronless ORF and cDNAs encoding the B-boxes and the first MATH domain can be found in the databases (Accession numbers FE580876.1 and FE556638.1). Also, as it will be detailed below, additional, very similar genes are found in *B. floridae*. It turns out that this gene does not belong to Subfamily A, but to Subfamily C. It is a false positive, detected due to structural convergence: two different acquisitions of MATH domains by unrelated TRIMs (see below).

### A Potential Evolutionary Link between TRIM and TRAF Ubiquitin Ligases

The finding of *TRIM37*-related genes in many eukaryotic groups has an important additional implication. It is now possible to hypothesize that the oldest TRIM genes, from which derive all the animal TRIMs, already contained MATH domains. If this is true, the MATH domain could be used as a marker to find other genes related to the ones in the TRIM family. There are only a few protein families, all of them eukaryotic, which contain MATH domains (reviewed in [85]). Among them, the most interesting, because of its structural and functional similarity with TRIMs, is the TNF Receptor-Associated Factor (TRAF) family of ubiquitin ligases [87], [88]. In parallel to what is found in Subfamily A TRIM proteins, most TRAF proteins contain an N-terminal RING finger and a C-terminal MATH domain. Between the RING and the MATH domains, just in the place where the B boxes typical of the TRIM family are, TRAF proteins contain 1 to 7 cysteine-rich domains similar to zinc fingers, but with a peculiar, TRAF-specific structure [87]–[90]. Given this structural similarity between Subfamily A TRIM proteins and TRAF proteins, it is logical to explore whether they may be evolutionary related. All TRAF proteins detected so far derive from animal genomes (from sponges to vertebrates, according to our observations). Therefore, it is possible to hypothesize that Subfamily A TRIM genes may be at the origin of not only all the rest of TRIMs but also of TRAF genes. If this is correct, we would expect to find the MATH domains of TRIM and TRAF genes to be related in sequence. When comprehensive phylogenetic analyses based on the protein sequences of MATH domains were performed, the domains in TRIM, TRAF and a third protein family, called Meprin (a peculiar kind of chordate-specific membrane peptidases), appeared together in all three types of phylogenetic analyses (NJ, MP and ML), albeit with low bootstrap support (Figure 2). These results support but do not fully demonstrate a relationship between the TRIM and TRAF E3s. However, they should encourage further research, especially considering the general lack of resolution of the dendrogram that relates all MATH domain-containing proteins (Figure 2). Even proteins that are clearly homologous in different groups of organisms (see e.g. the MATH BTB and MATH UCH groups of animals, plants or fungi) appeared either poorly supported or even separated in that dendrogram (Figure 2). This is caused by the short length of the MATH domain, about 100 amino acids long, which provides only limited information, precluding to obtain clearer results. In summary, it can be hypothesized from the available data that TRIMs and TRAFs are evolutionary related and that the MATH domains present in Meprins, which are intimately related to those of TRAFs ubiquitin ligases (Figure 2), were recently coopted, in the chordate lineage, from a TRAF protein.

A second significant finding, also shown in Figure 2, is that a few TRIMs found in a single species, *Branchiostoma floridae*, contain two MATH domains that are very different from the one present in TRIM Subfamily A sequences and very similar to those found in MATH UCH proteins (see “*Branchiostoma* TRIM” in that figure). One of these uncommon genes was already mentioned above; it was the only one encoding a protein with two B boxes that appeared in Figure 1. The other *Branchiostoma* genes were apparently too dissimilar as to be detected in the Tblastn searches from which the sequences in Figure 1 were obtained, all of them appearing together in other Tblastn searches, as typical Subfamily C TRIMs (not shown). Thus, the presence of TRIM and MATH domains in both all Subfamily A genes and these few *Branchiostoma*-specific Subfamily C genes must be due to structural convergence, caused by a recent recombinational event in the lineage that gave rise to *Branchiostoma*. Finally, notice the important fact that all the MATH domains present in TRIM proteins (but the few exceptional ones of Subfamily C just mentioned) appear together in the MATH-based phylogenetic analyses (Figure 2). This result reinforces the idea that all Subfamily A genes have a common, ancient origin, in agreement with the results of the Tblastn analyses and corresponding trees that were summarized in Figure 1.

## Discussion

The results described in the previous sections deepen our understanding of the origin, diversification and long-term evolution of the TRIM family. First, they provide a novel, more precise classification of TRIM proteins into natural groups, called subfamilies in this text, which is supported by sequence data and often also by structural data. This classification largely confirms the previous one based only in protein structures, but some significant differences, already mentioned in detail above, have been found. Also, these analyses invalidate the idea proposed by Sardiello *et al.* [23], which suggested that TRIMs were divided into just two groups, “Group 2” corresponding to our Subfamily G ( = Class IV in the structural classifications) and “Group 1” including all the rest. That this proposal is illogical is shown by the fact that at least three TRIM subfamilies (A–C), which include proteins with very different sequences and structures, were already present before animals emerged or very early in animal evolution (Table 1), while Subfamily G is just a very recently emerged group, restricted to vertebrates. Thus, the classification into just two groups is not supported by evolutionary data. Actually, the evidence obtained to separate all TRIMs into those two groups was quite weak. It consisted in: 1) an unrooted phylogenetic tree of human TRIMs, without any bootstrap analysis to support its topology. and, 2) some additional data showing that the genomic organization of the genes in “Group 2” is quite homogeneous while “Group 1” genes are much more diverse [23]. However, both the fact that Subfamily G ( = “Group 2”) genes appear together in that tree and their genomic similarity can be simply explained by the recent origin of all genes of this subfamily.

A second significant result obtained is that six vertebrate genes generally considered unrelated to TRIM genes in fact encode TRIM-like proteins, with significant similarity to canonical TRIMs (Table 1). These genes (*RNF207*, *FSD1*, *FSD2*, *FSD1L*, *NHLCR1/Malin* and *BSPRY*) arose by duplications of typical TRIMs followed by deletions that eliminated regions encoding part of the RBCC signature. Among them, perhaps the best known is *NHLRC1/Malin*. Mutations in this gene cause Lafora’s disease, a neurodegenerative disorder (reviewed in [91]). Malin is a short protein with a RING finger and six NHL repeats that lacks any B boxes, explaining why it was not classified as a TRIM protein. However, as simple Blast searches demonstrate, Malin NHL repeats are very similar to those found in typical Subfamily C TRIMs (see also [92]). *RNF207* was also considered a TRIM-encoding gene in a recent study [25].

A third important result is that evidence for TRIM genes to be much more ancient than previously thought has been obtained (Table 1 and Figure 1). These *TRIM37*-related genes have been missed before because they are present in just a few, recently sequenced species. The patchy pattern of presence/absence of the *TRIM37*-related genes can be explained by multiple gene losses. That *TRIM37* genes are often lost is supported by the lack of these genes in model species such as *D. melanogaster* or *C. elegans* in spite of being present in related species. The only alternative explanation for their patchy phylogenetic range would require invoking horizontal transmission among distant eukaryotes. This is a theoretically possible but quite far-fetched explanation, especially given the wide range of organisms that contain these genes. First, to support this idea, many independent horizontal transmissions of *TRIM37* genes to totally unrelated organisms must be postulated, which seems very unlikely. Second, even accepting that uncommon horizontal transmissions of *TRIM* genes among very distant organisms may occur, postulating that *TRIM37* has been the only one systematically involved in this type of process, while none of the other *TRIM* genes have been horizontally transferred, is also difficult to accept.

The fourth main result derives from the fact that these ancient TRIM genes encode proteins with MATH domains. This suggests that there may be an evolutionary link between TRIM and TRAF ubiquitin ligases, given the presence in both families of similar structures (RING+MATH) that may have a common origin (Figure 2). Postulating a common origin of TRIM and TRAF ubiquitin ligases fits well with the fact that some of them have related roles. For example, the Subfamily F protein TRIM25/EFP is known to act as part of the innate immune response linked to interferon production, in a way that resembles how several TRAFs function [58]. Also, both TRIM25/EFP and TRAF6 are able to generate free ubiquitin chains (i. e. chains not attached to other proteins) that act as scaffolds, favoring interactions among other proteins, which are critical for the interferon-induced response [93]–[95]. Future research may determine whether these links are more profound than so far established.

Some minor results, as the discovery of structurally convergent TRIM proteins, i. e. proteins which have similar structures due to independent acquisitions of a same protein domain, are also interesting. This result highlights why the classification of a complex family into natural groups or subfamilies improves our ability to interpret functional data. Many discussions are flawed by not considering whether similarities among proteins are either due to common origin or to convergence. A typical example regards HERC ubiquitin ligases, a type of HECT E3s. Generally discussed as a single family [96], we recently showed that there are indeed two different groups of HERCs, which originated independently [8]. A second typical example is the convergent similarity of the Parkinson disease-related E3 parkin and another protein of the RBR family, called RBCK1 (a. k. a. XAP3, HOIL), which both have ubiquitin-like domains [12]. The presence, described above, of a few *Branchiostoma* TRIMs that resemble *TRIM37* due to convergence is a similar finding. Classification errors due to convergence associated to independent losses of protein domains are also possible, as the results showed above for the inexistent “Class V” [20], [77] show.

Some functional hypotheses can be formulated thanks to the information described in this study. For example, it seems logical to hypothesize that the oldest TRIM proteins most likely worked already as ubiquitin ligases. This is suggested by many proteins of all subfamilies, including the ancient Subfamily A, having that biochemical function. On the contrary, the ability of a few TRIMs to act as SUMO ligases [76] seems to have emerged more recently and probably several times independently. Also, so far only a single TRIM, TRIM25/EFP, is known to be able to ISGylate [74]. This is most likely also a recent novelty, especially given that this same protein may also act as ubiquitin ligase [58]. A second logical hypothesis to put forward is that perhaps the most ancient functions of TRIM proteins in animal species were very general, perhaps housekeeping roles in multiple tissues or cell types. This can be suggested based on the fact that mutations in the gene encoding a Subfamily A protein, human *TRIM37,* cause mulibrey nanism, a syndrome with multiple development failures (growth retardation, damages in heart, muscle, liver, brain, etc.; reviewed in [97]). A third hypothesis that deserves further exploration concerns the roles of TRIM proteins in innate immunity. The available data suggest that TRIM proteins have been coopted multiple times independently to perform those roles, probably later than their original, more “internal” roles. This hypothesis is based on the fact that multiple proteins of different subfamilies are known to be linked to innate immunity (such as TRIM56 - Subfamily B; TRIM23/ARD1, TRIM28/TIF1-beta – Subfamily D; TRIM5, TRIM11, TRIM22/STAF50, TRIM25/EFP – Subfamily F and TRIM19/PML – Subfamily L), but apparently not those in Subfamily A, such as TRIM37. Interestingly, this potential dichotomy between conventional roles, often housekeeping, as part of the cell internal ubiquitination system and more recent roles in innate inmunity has been already hypothesized for other types of ubiquitin ligases, such as RBR E3s [15]. It is significant that rapid expansions of very similar sets of genes generated by tandem gene duplications are detectable in both animal TRIM E3s and some RBR E3s. These expansions may be related to the ability to respond to external aggressions. Regarding the TRIM family, this is an interesting topic that we plan to address in detail in future works.

## References

[1] Glickman MH, Ciechanover A (2002) The ubiquitin-proteasome proteolytic pathway: destruction for the sake of construction. Physiol Rev 82: 373–428. 11917093 10.1152/physrev.00027.2001

[2] Kerscher O, Felberbaum R, Hochstrasser M (2006) Modification of proteins by ubiquitin and ubiquitin-like. Annu Rev Cell Dev Biol 22: 159–180. 16753028 10.1146/annurev.cellbio.22.010605.093503

[3] Chen ZJ, Sun LJ (2009) Nonproteolytic functions of ubiquitin in cell signaling. Mol Cell 33: 275–286. 19217402 10.1016/j.molcel.2009.01.014

[4] Komander D (2009) The emerging complexity of protein ubiquitination. Biochem Soc Trans 37: 937–953. 19754430 10.1042/BST0370937

[5] Schwartz AL, Ciechanover A (2009) Targeting proteins for destruction by the ubiquitin system: implications for human pathobiology. Annu Rev Pharmacol Toxicol 49: 73–96. 18834306 10.1146/annurev.pharmtox.051208.165340

[6] Behrends C, Harper JW (2011) Constructing and decoding unconventional ubiquitin chains. Nat Struct Mol Biol 18: 520–528. 21540891 10.1038/nsmb.2066

[7] Marín I (2009) Diversification of the cullin family. BMC Evol Biol 9: 267. 19925652 10.1186/1471-2148-9-267 PMC2785787

[8] Marín I (2010) Animal HECT ubiquitin ligases: evolution and functional implications. BMC Evol Biol 10: 56. 20175895 10.1186/1471-2148-10-56 PMC2837046

[9] Marín I (2010) Ancient origin of animal U-box ubiquitin ligases. BMC Evol Biol 10: 331. 20979629 10.1186/1471-2148-10-331 PMC3087547

[10] Deshaies RJ, Joazeiro CA (2009) RING domain E3 ubiquitin ligases. Annu Rev Biochem 78: 399–434. 19489725 10.1146/annurev.biochem.78.101807.093809

[11] Marín I, Lucas JI, Gradilla AC, Ferrús A (2004) Parkin and relatives: the RBR family of ubiquitin ligases. Physiol Genomics 17: 253–263. 15152079 10.1152/physiolgenomics.00226.2003

[12] Marín I, Ferrús A (2002) Comparative genomics of the RBR family, including the Parkinson’s disease-related gene *parkin* and the genes of the ariadne subfamily. Mol Biol Evol 19: 2039–2050. 12446796 10.1093/oxfordjournals.molbev.a004029

[13] Lucas JI, Arnau V, Marín I (2006) Comparative genomics and protein domain graph analyses link ubiquitination and RNA metabolism. J Mol Biol 357: 9–17. 16426638 10.1016/j.jmb.2005.12.068

[14] Marín I (2009) RBR ubiquitin ligases: Diversification and streamlining in animal lineages. J Mol Evol 69: 54–64. 19526189 10.1007/s00239-009-9252-3

[15] Marín I (2010) Diversification and specialization of plant RBR ubiquitin ligases. PLoS One 5: e11579. 20644651 10.1371/journal.pone.0011579 PMC2904391

[16] Wenzel DM, Lissounov A, Brzovic PS, Klevit RE (2011) UBCH7 reactivity profile reveals parkin and HHARI to be RING/HECT hybrids. Nature 474: 105–108. 21532592 10.1038/nature09966 PMC3444301

[17] Torok M, Etkin LD (2001) Two B or not two B? Overview of the rapidly expanding B-box family of proteins. Differentiation 67: 63–71. 11428128 10.1046/j.1432-0436.2001.067003063.x

[18] Meroni G, Diez-Roux G (2005) TRIM/RBCC, a novel class of ‘single protein RING finger’ E3 ubiquitin ligases. Bioessays 27: 1147–1157. 16237670 10.1002/bies.20304

[19] Nisole S, Stoye JP, Saïb A (2005) TRIM family proteins: retroviral restriction and antiviral defence. Nat Rev Microbiol 3: 799–808. 16175175 10.1038/nrmicro1248

[20] Ozato K, Shin DM, Chang TH, Morse HC 3^rd^ (2008) TRIM family proteins and their emerging roles in innate immunity. Nat Rev Immunol 8: 849–860. 18836477 10.1038/nri2413 PMC3433745

[21] McNab FW, Rajsbaum R, Stoye JP, O’Garra A (2011) Tripartite-motif proteins and innate immune regulation. Curr Opin Immunol 23: 46–56. 21131187 10.1016/j.coi.2010.10.021

[22] Massiah MA, Matts JA, Short KM, Simmons BN, Singireddy S, et al (2007) Solution structure of the MID1 B-box2 CHC(D/C)C(2)H(2) zinc-binding domain: insights into an evolutionarily conserved RING fold. J Mol Biol 369: 1–10. 17428496 10.1016/j.jmb.2007.03.017

[23] Sardiello M, Cairo S, Fontanella B, Ballabio A, Meroni G (2008) Genomic analysis of the TRIM family reveals two groups of genes with distinct evolutionary properties. BMC Evol Biol 8: 225. 18673550 10.1186/1471-2148-8-225 PMC2533329

[24] van der Aa LM, Levraud JP, Yahmi M, Lauret E, Briolat V, et al (2009) A large new subset of TRIM genes highly diversified by duplication and positive selection in teleost fish. BMC Biol 7: 7. 19196451 10.1186/1741-7007-7-7 PMC2657112

[25] Boudinot P, van der Aa LM, Jouneau L, Du Pasquier L, Pontarotti P, et al (2011) Origin and evolution of TRIM proteins: new insights from the complete TRIM repertoire of zebrafish and pufferfish. PLoS One 6: e22022. 21789205 10.1371/journal.pone.0022022 PMC3137616

[26] Tae H, Casarotto MG, Dulhunty AF (2009) Ubiquitous SPRY domains and their role in the skeletal type ryanodine receptor. Eur Biophys J 39: 51–59. 19399493 10.1007/s00249-009-0455-8

[27] Du Pasquier L (2009) Fish ‘n’ TRIMs. J Biol 8: 50. 19519941 10.1186/jbiol150 PMC2736664

[28] Avela K, Lipsanen-Nyman M, Idänheimo N, Seemanová E, Rosengren S, et al (2000) Gene encoding a new RING-B-box-Coiled-coil protein is mutated in mulibrey nanism. Nat Genet 25: 298–301. 10888877 10.1038/77053

[29] Quaderi NA, Schweiger S, Gaudenz K, Franco B, Rugarli EI, et al (1997) Opitz G/BBB syndrome, a defect of midline development, is due to mutations in a new RING finger gene on Xp22. Nat Genet 17: 285–291. 9354791 10.1038/ng1197-285

[30] Frosk P, Weiler T, Nylen E, Sudha T, Greenberg CR, et al (2002) Limb-girdle muscular dystrophy type 2H associated with mutation in *TRIM32*, a putative E3-ubiquitin-ligase gene. Am J Hum Genet 70: 663–672. 11822024 10.1086/339083 PMC447621

[31] Chiang AP, Beck JS, Yen HJ, Tayeh MK, Scheetz TE, et al (2006) Homozygosity mapping with SNP arrays identifies TRIM32, an E3 ubiquitin ligase, as a Bardet-Biedl syndrome gene (*BBS11*). Proc Natl Acad Sci U S A 103: 6287–6292. 16606853 10.1073/pnas.0600158103 PMC1458870

[32] Saccone V, Palmieri M, Passamano L, Piluso G, Meroni G, et al (2008) Mutations that impair interaction properties of TRIM32 associated with limb-girdle muscular dystrophy 2H. Hum Mutat 29: 240–247. 17994549 10.1002/humu.20633

[33] French FMF Consortium (1997) A candidate gene for familial Mediterranean fever. Nat Genet 17: 25–31. 9288094 10.1038/ng0997-25

[34] The International FMF Consortium (1997) Ancient missense mutations in a new member of the RoRet gene family are likely to cause familial Mediterranean fever. Cell 90: 797–807. 9288758 10.1016/s0092-8674(00)80539-5

[35] Bernot A, da Silva C, Petit JL, Cruaud C, Caloustian C, et al (1998) Non-founder mutations in the *MEFV* gene establish this gene as the cause of familial Mediterranean fever (FMF). Hum Mol Genet 7: 1317–1325. 9668175 10.1093/hmg/7.8.1317

[36] Keech CL, Howarth S, Coates T, Rischmueller M, McCluskey J, et al (1996) Rapid and sensitive detection of anti-Ro (SS-A) antibodies by indirect immunofluorescence of 60 kDa Ro HEp-2 transfectants. Pathology 28: 54–57. 8714273 10.1080/00313029600169533

[37] Salomonsson S, Sonesson SE, Ottosson L, Muhallab S, Olsson T, et al (2005) Ro/SSA autoantibodies directly bind cardiomyocytes, disturb calcium homeostasis, and mediate congenital heart block. J Exp Med 201: 11–17. 15630133 10.1084/jem.20041859 PMC2212767

[38] Kurata R, Nakaoka H, Tajima A, Hosomichi K, Shiina T, et al (2010) TRIM39 and RNF39 are associated with Behçet’s disease independently of HLA-B^∗^51 and -A^∗^26. Biochem Biophys Res Commun 401: 533–537. 20875797 10.1016/j.bbrc.2010.09.088

[39] Reynolds JG, McCalmon SA, Donaghey JA, Naya FJ (2008) Deregulated protein kinase A signaling and myospryn expression in muscular dystrophy. J Biol Chem 283: 8070–8074. 18252718 10.1074/jbc.C700221200 PMC2276392

[40] Balastik M, Ferraguti F, Pires-da Silva A, Lee TH, Alvarez-Bolado G, et al (2008) Deficiency in ubiquitin ligase TRIM2 causes accumulation of neurofilament light chain and neurodegeneration. Proc Natl Acad Sci U S A 105: 12016–12021. 18687884 10.1073/pnas.0802261105 PMC2575299

[41] Shi H, Medway C, Bullock J, Brown K, Kalsheker N, et al (2010) Analysis of Genome-Wide Association Study (GWAS) data looking for replicating signals in Alzheimer’s disease (AD). Int J Mol Epidemiol Genet 1: 53–66. 21537453 PMC3076755

[42] Takahashi M, Buma Y, Iwamoto T, Inaguma Y, Ikeda H, et al (1988) Cloning and expression of the ret proto-oncogene encoding a tyrosine kinase with two potential transmembrane domains. Oncogene 3: 571–578. 3078962

[43] Le Douarin B, Zechel C, Garnier JM, Lutz Y, Tora L, et al (1995) The N-terminal part of TIF1, a putative mediator of the ligand-dependent activation function (AF-2) of nuclear receptors, is fused to B-raf in the oncogenic protein T18. EMBO J 14: 2020–2033. 7744009 10.1002/j.1460-2075.1995.tb07194.x PMC398302

[44] Corcoran MM, Hammarsund M, Zhu C, Lerner M, Kapanadze B, et al (2004) DLEU2 encodes an antisense RNA for the putative bicistronic *RFP2/LEU5* gene in humans and mouse. Genes Chromosomes Cancer 40: 285–297. 15188451 10.1002/gcc.20046

[45] Zirn B, Hartmann O, Samans B, Krause M, Wittmann S, et al (2006) Expression profiling of Wilms tumors reveals new candidate genes for different clinical parameters. Int J Cancer 118: 1954–1962. 16287080 10.1002/ijc.21564

[46] Lerner M, Corcoran M, Cepeda D, Nielsen ML, Zubarev R, et al (2007) The RBCC gene *RFP2* (*Leu5*) encodes a novel transmembrane E3 ubiquitin ligase involved in ERAD. Mol Biol Cell 18: 1670–1682. 17314412 10.1091/mbc.E06-03-0248 PMC1855009

[47] Karlberg S, Lipsanen-Nyman M, Lassus H, Kallijärvi J, Lehesjoki AE, et al (2009) Gynecological tumors in Mulibrey nanism and role for RING finger protein TRIM37 in the pathogenesis of ovarian fibrothecomas. Mod Pathol 22: 570–578. 19329943 10.1038/modpathol.2009.13

[48] Lott ST, Chen N, Chandler DS, Yang Q, Wang L, et al (2009) DEAR1 is a dominant regulator of acinar morphogenesis and an independent predictor of local recurrence-free survival in early-onset breast cancer. PLoS Med 6: e1000068. 19536326 10.1371/journal.pmed.1000068 PMC2673042

[49] Wang L, Heidt DG, Lee CJ, Yang H, Logsdon CD, et al (2009) Oncogenic function of ATDC in pancreatic cancer through Wnt pathway activation and beta-catenin stabilization. Cancer Cell 15: 207–219. 19249679 10.1016/j.ccr.2009.01.018 PMC2673547

[50] Hatakeyama S (2011) TRIM proteins and cancer. Nat Rev Cancer 11: 792–804. 21979307 10.1038/nrc3139

[51] Chelbi-Alix MK, Quignon F, Pelicano L, Koken MH, de Thé H (1998) Resistance to virus infection conferred by the interferon-induced promyelocytic leukemia protein. J Virol 72: 1043–1051. 9444998 10.1128/jvi.72.2.1043-1051.1998 PMC124576

[52] Stremlau M, Owens CM, Perron MJ, Kiessling M, Autissier P, et al (2004) The cytoplasmic body component TRIM5alpha restricts HIV-1 infection in Old World monkeys. Nature 427: 848–853. 14985764 10.1038/nature02343

[53] Yap MW, Nisole S, Lynch C, Stoye JP (2004) Trim5alpha protein restricts both HIV-1 and murine leukemia virus. Proc Natl Acad Sci U S A 101: 10786–10791. 15249690 10.1073/pnas.0402876101 PMC490012

[54] Perron MJ, Stremlau M, Song B, Ulm W, Mulligan RC, et al (2004) TRIM5alpha mediates the postentry block to N-tropic murine leukemia viruses in human cells. Proc Natl Acad Sci U S A 101: 11827–11832. 15280539 10.1073/pnas.0403364101 PMC511059

[55] Bouazzaoui A, Kreutz M, Eisert V, Dinauer N, Heinzelmann A, et al (2006) Stimulated trans-acting factor of 50 kDa (Staf50) inhibits HIV-1 replication in human monocyte-derived macrophages. Virology 356: 79–94. 16926043 10.1016/j.virol.2006.07.025

[56] Everett RD, Rechter S, Papior P, Tavalai N, Stamminger T, et al (2006) PML contributes to a cellular mechanism of repression of herpes simplex virus type 1 infection that is inactivated by ICP0. J Virol 80: 7995–8005. 16873256 10.1128/JVI.00734-06 PMC1563828

[57] Tavalai N, Papior P, Rechter S, Leis M, Stamminger T (2006) Evidence for a role of the cellular ND10 protein PML in mediating intrinsic immunity against human cytomegalovirus infections. J Virol 80: 8006–8018. 16873257 10.1128/JVI.00743-06 PMC1563799

[58] Gack MU, Shin YC, Joo CH, Urano T, Liang C, et al (2007) TRIM25 RING-finger E3 ubiquitin ligase is essential for RIG-I-mediated antiviral activity. Nature 446: 916–920. 17392790 10.1038/nature05732

[59] Gack MU, Kirchhofer A, Shin YC, Inn KS, Liang C, et al (2008) Roles of RIG-I N-terminal tandem CARD and splice variant in TRIM25-mediated antiviral signal transduction. Proc Natl Acad Sci U S A 105: 16743–16748. 18948594 10.1073/pnas.0804947105 PMC2575490

[60] Wolf D, Goff SP (2007) TRIM28 mediates primer binding site-targeted silencing of murine leukemia virus in embryonic cells. Cell 131: 46–57. 17923087 10.1016/j.cell.2007.07.026

[61] Ammermann I, Bruckner M, Matthes F, Iftner T, Stubenrauch F (2008) Inhibition of transcription and DNA replication by the papillomavirus E8-E2C protein is mediated by interaction with corepressor molecules. J Virol 82: 5127–5136. 18353941 10.1128/JVI.02647-07 PMC2395219

[62] Wolf D, Hug K, Goff SP (2008) TRIM28 mediates primer binding site-targeted silencing of Lys1,2 tRNA-utilizing retroviruses in embryonic cells. Proc Natl Acad Sci U S A 105: 12521–12526. 18713861 10.1073/pnas.0805540105 PMC2518094

[63] Yap MW, Lindemann D, Stanke N, Reh J, Westphal D, et al (2008) Restriction of foamy viruses by primate Trim5alpha. J Virol 82: 5429–5439. 18367529 10.1128/JVI.02462-07 PMC2395188

[64] Uchil PD, Quinlan BD, Chan WT, Luna JM, Mothes W (2008) TRIM E3 ligases interfere with early and late stages of the retroviral life cycle. PLoS Pathog 4: e16. 18248090 10.1371/journal.ppat.0040016 PMC2222954

[65] Chang PC, Fitzgerald LD, Van Geelen A, Izumiya Y, Ellison TJ, et al (2009) Kruppel-associated box domain-associated protein-1 as a latency regulator for Kaposi’s sarcoma-associated herpesvirus and its modulation by the viral protein kinase. Cancer Res 69: 5681–5689. 19584288 10.1158/0008-5472.CAN-08-4570 PMC2731626

[66] Eldin P, Papon L, Oteiza A, Brocchi E, Lawson TG, et al (2009) TRIM22 E3 ubiquitin ligase activity is required to mediate antiviral activity against encephalomyocarditis virus. J Gen Virol 90: 536–545. 19218198 10.1099/vir.0.006288-0

[67] Gao B, Duan Z, Xu W, Xiong S (2009) Tripartite motif-containing 22 inhibits the activity of hepatitis B virus core promoter, which is dependent on nuclear-located RING domain. Hepatology 50: 424–433. 19585648 10.1002/hep.23011

[68] Wolf D, Goff SP (2009) Embryonic stem cells use ZFP809 to silence retroviral DNAs. Nature 458: 1201–1204. 19270682 10.1038/nature07844 PMC2676211

[69] Poole E, Groves I, MacDonald A, Pang Y, Alcami A, et al (2009) Identification of TRIM23 as a cofactor involved in the regulation of NF-kappaB by human cytomegalovirus. J Virol 83: 3581–3590. 19176615 10.1128/JVI.02072-08 PMC2663253

[70] Arimoto K, Funami K, Saeki Y, Tanaka K, Okawa K, et al (2010) Polyubiquitin conjugation to NEMO by triparite motif protein 23 (TRIM23) is critical in antiviral defense. Proc Natl Acad Sci U S A 107: 15856–15861. 20724660 10.1073/pnas.1004621107 PMC2936632

[71] Kajaste-Rudnitski A, Marelli SS, Pultrone C, Pertel T, Uchil PD, et al (2011) TRIM22 inhibits HIV-1 transcription independently of its E3 ubiquitin ligase activity, Tat, and NF-kappaB-responsive long terminal repeat elements. J Virol 85: 5183–5196. 21345949 10.1128/JVI.02302-10 PMC3126207

[72] Taylor RT, Lubick KJ, Robertson SJ, Broughton JP, Bloom ME, et al (2011) TRIM79α, an interferon-stimulated gene product, restricts tick-borne encephalitis virus replication by degrading the viral RNA polymerase. Cell Host Microbe 10: 185–196. 21925107 10.1016/j.chom.2011.08.004 PMC3182769

[73] Wang J, Liu B, Wang N, Lee YM, Liu C, et al (2011) TRIM56 is a virus- and interferon-inducible E3 ubiquitin ligase that restricts pestivirus infection. J Virol 85: 3733–3745. 21289118 10.1128/JVI.02546-10 PMC3126137

[74] Zou W, Zhang DE (2006) The interferon-inducible ubiquitin-protein isopeptide ligase (E3) EFP also functions as an ISG15 E3 ligase. J Biol Chem 281: 3989–3994. 16352599 10.1074/jbc.M510787200

[75] Duprez E, Saurin AJ, Desterro JM, Lallemand-Breitenbach V, Howe K, et al (1999) SUMO-1 modification of the acute promyelocytic leukaemia protein PML: implications for nuclear localisation. J Cell Sci 112: 381–393. 9885291 10.1242/jcs.112.3.381

[76] Chu Y, Yang X (2011) SUMO E3 ligase activity of TRIM proteins. Oncogene 30: 1108–1116. 20972456 10.1038/onc.2010.462 PMC3103664

[77] Short KM, Cox TC (2006) Subclassification of the RBCC/TRIM superfamily reveals a novel motif necessary for microtubule binding. J Biol Chem 281: 8970–8980. 16434393 10.1074/jbc.M512755200

[78] Larkin MA, Blackshields G, Brown NP, Chenna R, McGettigan PA, et al (2007) Clustal W and Clustal X version 2.0. Bioinformatics 23: 2947–2948. 17846036 10.1093/bioinformatics/btm404

[79] Katoh K, Toh H (2008) Recent developments in the MAFFT multiple sequence alignment program. Briefings Bioinf 9: 286–298. 10.1093/bib/bbn013 18372315

[80] Nicholas KB, Nicholas HB Jr (1997) GeneDoc: a tool for editing and annotating multiple sequence alignments. Distributed by the authors.

[81] Tamura K, Peterson D, Peterson N, Stecher G, Nei M, et al (2011) MEGA5: Molecular Evolutionary Genetics Analysis Using Maximum Likelihood, Evolutionary Distance, and Maximum Parsimony Methods. Mol Biol Evol 28: 2731–2739. 21546353 10.1093/molbev/msr121 PMC3203626

[82] Swofford DL (2003) PAUP*. Phylogenetic Analysis Using Parsimony (* and Other Methods). Version 4. Sinauer Associates, Sunderland, Massachusetts.

[83] Dwivedi B, Gadagkar SR (2009) Phylogenetic inference under varying proportions of indel-induced alignment gaps. BMC Evol Biol 9: 211. 19698168 10.1186/1471-2148-9-211 PMC2746219

[84] Zdobnov EM, Apweiler R (2001) InterProScan - an integration platform for the signature-recognition methods in InterPro. Bioinformatics 17: 847–848. 11590104 10.1093/bioinformatics/17.9.847

[85] Zapata JM, Martínez-García V, Lefebvre S (2007) Phylogeny of the TRAF/MATH domain. Adv Exp Med Biol 597: 1–24. 17633013 10.1007/978-0-387-70630-6_1

[86] Ye H, Park YC, Kreishman M, Kieff E, Wu H (1999) The structural basis for the recognition of diverse receptor sequences by TRAF2. Mol Cell 4: 321–330. 10518213 10.1016/s1097-2765(00)80334-2

[87] Bradley JR, Pober JS (2001) Tumor necrosis factor receptor-associated factors (TRAFs). Oncogene 20: 6482–6491. 11607847 10.1038/sj.onc.1204788

[88] Wajant H, Henkler F, Scheurich P (2001) The TNF-receptor-associated factor family: scaffold molecules for cytokine receptors, kinases and their regulators. Cell Signal 13: 389–400. 11384837 10.1016/s0898-6568(01)00160-7

[89] Chung JY, Park YC, Ye H, Wu H (2002) All TRAFs are not created equal: common and distinct molecular mechanisms of TRAF-mediated signal transduction. J Cell Sci 115: 679–688. 11865024 10.1242/jcs.115.4.679

[90] Pineda G, Ea CK, Chen ZJ (2007) Ubiquitination and TRAF signaling. Adv Exp Med Biol 597: 80–92. 17633019 10.1007/978-0-387-70630-6_7

[91] Gentry MS, Dixon JE, Worby CA (2009) Lafora disease: insights into neurodegeneration from plant metabolism. Trends Biochem Sci 34: 628–639. 19818631 10.1016/j.tibs.2009.08.002 PMC2805077

[92] Romá-Mateo C, Moreno D, Vernia S, Rubio T, Bridges TM, et al (2011) Lafora disease E3-ubiquitin ligase malin is related to TRIM32 at both the phylogenetic and functional level. BMC Evol Biol 11: 225. 21798009 10.1186/1471-2148-11-225 PMC3160408

[93] Deng L, Wang C, Spencer E, Yang L, Braun A, et al (2000) Activation of the IkappaB kinase complex by TRAF6 requires a dimeric ubiquitin-conjugating enzyme complex and a unique polyubiquitin chain. Cell 103: 351–361. 11057907 10.1016/s0092-8674(00)00126-4

[94] Xia ZP, Sun L, Chen X, Pineda G, Jiang X, et al (2009) Direct activation of protein kinases by unanchored polyubiquitin chains. Nature 461: 114–119. 19675569 10.1038/nature08247 PMC2747300

[95] Zeng W, Sun L, Jiang X, Chen X, Hou F, et al (2010) Reconstitution of the RIG-I pathway reveals a signaling role of unanchored polyubiquitin chains in innate immunity. Cell 141: 315–330. 20403326 10.1016/j.cell.2010.03.029 PMC2919214

[96] Garcia-Gonzalo FR, Rosa JL (2005) The HERC proteins: functional and evolutionary insights. Cell Mol Life Sci 62: 1826–1838. 15968461 10.1007/s00018-005-5119-y PMC11139159

[97] Hes FJ, Morreau H (2009) Where genetics and pathology meet: mulibrey nanism. J Pathol 218: 143–145. 19347900 10.1002/path.2552

